# RNA Sequencing Identified Differentially Expressed Genes in the Mesocorticolimbic and Nigrostriatal Systems of Compulsive METH-Taking Rats

**DOI:** 10.3390/cells14181472

**Published:** 2025-09-20

**Authors:** Nasser Adjei, Bruce Ladenheim, Michael T. McCoy, Vikrant Palande, Jean Lud Cadet, Atul P. Daiwile

**Affiliations:** 1 Molecular Neuropsychiatry Research Branch, NIDA Intramural Research Program, Baltimore, MD 21224, USA; nasser.adjei@nih.gov (N.A.); bladen@intra.nida.nih.gov (B.L.); mmccoy@intra.nida.nih.gov (M.T.M.); jcadet@intra.nida.nih.gov (J.L.C.); 2 Department of Pathology, Johns Hopkins University, Baltimore, MD 21287, USA; vpaland1@jhu.edu

**Keywords:** methamphetamine self-administration, foot-shocks, RNA sequencing, prefrontal cortex, nucleus accumbens, dorsal striatum, midbrain

## Abstract

Methamphetamine (METH) is an extremely addictive drug which continues to cause significant harm to individuals and communities. In the present study we trained male rats to self-administer METH for 20 days, followed by 9 days of foot shock exposure. All rats escalated their METH intake during the first 20 days. The rats that continued to self-administer METH in the presence of aversive stimuli were termed shock-resistant (SR), while those that reduced their intake were shock-sensitive (SS). RNA sequencing showed numerous differentially expressed genes (DEGs) in the prefrontal cortex, nucleus accumbens, dorsal striatum, and midbrain. Ingenuity pathway analysis linked DEGs to addiction-related mechanisms. We identified shared genes with similar expression patterns across four brain regions (SR: *Fos* and *Ahsp*; SS: *Tet1*, *Cym*, and *Tmem30c*). The identified genes play key roles in addiction-related brain functions, such as neuronal activity, stress response, and epigenetic regulation, and their importance in METH addiction is highlighted. These genes represent promising targets for developing new treatments aimed at reversing neuroadaptations caused by METH use.

## 1. Introduction

Methamphetamine (METH) is a highly addictive amphetamine-type psychostimulant with a growing prevalence of misuse worldwide [1,2,3]. METH has risen in popularity for inducing feelings of euphoria, increased productivity, and heightened energy [1,2,3]. The half-life of METH ranges from 10 h to 12 h, allowing for its effects to persist for a prolonged period [4]. It can be taken orally, snorted, smoked, or injected intravenously. Its effects are mediated through the release and changes in the metabolism of monoamines, such as dopamine and noradrenaline, from synaptic vesicles [1,2,3].

From 2015 to 2019, overdose deaths in the United States involving psychostimulants excluding cocaine increased rapidly, with methamphetamine being the primary contributor [5]. During this period, methamphetamine-related overdose deaths involving fentanyl also rose sharply, from 7% to 31%. Although mortality rates have significantly increased, the rates of methamphetamine use disorder (MUD) have shown only a slight, insignificant rise. This suggests that the heightened overdose risk among methamphetamine users is largely driven by fentanyl contamination [5]. It is important to note that while many methamphetamine users meet the DSM-V criteria for MUD, not all do [6]. MUD is characterized by behaviors of excessive drug-taking during binge periods, continued use despite adverse consequences, and craving drugs [6].

Methamphetamine use disorder (MUD) is associated with several negative consequences in humans, including acute toxicity, neurological damage, and altered behavioral and cognitive functions [2,3]. When taken at high doses, METH can cause life-threatening outcomes such as hyperthermia above 41 °C, cardiac arrhythmias, heart attacks, cerebrovascular hemorrhages, strokes, seizures, and failure of the kidneys and liver [7,8]. Prolonged abuse of METH is known to contribute to anxiety, depression, aggressiveness, social isolation, psychosis, mood disturbances, and psychomotor dysfunction [3,6,7]. Neuropsychological studies have detected deficits in attention, working memory, and decision-making in chronic METH users [2,3]. There is compelling evidence that the negative neuropsychiatric consequences of METH abuse are related to neuroadaptive changes in brain regions responsible for the rewarding effects of drugs and the cognitive processes that regulate learned habitual behaviors [9,10]. These neuroadaptations are believed to involve altered synaptic plasticity, transcriptional alterations, and epigenetic changes in the nigrostriatal and mesocorticolimbic projection areas [2,3,11,12].

The prefrontal cortex (PFC), nucleus accumbens (NAc), dorsal striatum (DStr), and midbrain (MBr) form the neural network that regulates reward, habit formation, motivation, and decision-making [13]. The ventral tegmental area and substantia nigra located in the MBr contain dopamine neurons, which are activated in response to rewarding stimuli. Dopamine released during these events propagates to the NAc, an important region of the reward circuit, reinforcing the pleasurable effects and promoting drug-taking behavior [13,14]. As addiction progresses, control shifts to the DStr, which is involved in habit formation, causing compulsive drug use [15]. The PFC responsible for executive functions, decision-making, and impulse control becomes impaired, leading to a reduced ability to regulate drug-taking behaviors. The PFC’s weakened control over the NAc and DStr exacerbates addiction as the brain reward and habit system dominate, overriding rational decision-making [13,14,16].

To develop more efficacious approaches for METH addiction treatment, it is crucial to elucidate the neurobiological substrates of acquisition and persistent abuse of the drug. Rat self-administration models are often used to study potential molecular bases of drug-taking behaviors by humans [17]. METH self-administering rats that are given extended access to the drug escalate its use over time [12]. Drug models of METH self-administration have been instrumental in identifying the potential roles of various neurotransmitter systems, as well as the involvement of gene regulation and epigenetic mechanisms in modulating METH-taking behaviors [12,18,19]. To investigate the molecular bases of compulsive behaviors seen in methamphetamine use disorder (MUD), our lab modeled the DSM-V criterion of compulsive use despite adverse consequences by applying foot shocks contingently during METH self-administration. Punishment is crucial in this model as it allows the separation of rats into compulsive and non-compulsive METH takers [20]. The present study was carried out to identify transcriptional changes in the PFC, NAc, DStr, and MBr of rats that exhibited two distinct phenotypes of drug-taking behavior while receiving contingent foot shocks during METH self-administration. One group of animals continued to compulsively press the lever to receive the drug (shock-resistant), whereas the other reduced their drug intake (shock-sensitive).

## 2. Materials and Methods

### 2.1. Animals

Male Sprague-Dawley rats weighing 350–400 g were purchased from Charles River, USA. The rats were housed in a controlled setting with a reversed 12 h light/dark cycle with free access to food and water. All animal procedures were approved by the National Institute of Drug Abuse Animal Care and Use Committee (Protocol No. 24-MNPB-9) and conducted according to the Guide for the Care and Use of Laboratory Animals (ISBN 0-309-05377-3).

### 2.2. Intravenous Surgery

The rat intravenous catheter surgery was performed according to previously published protocols [20,21]. Briefly, rats were anesthetized with a combination of ketamine (NIDA Drug Supply, Baltimore, MD, USA) and xylazine (NIDA Drug Supply, Baltimore, MD, USA) at dosages of 50 mg/kg and 5 mg/kg, respectively. A polyurethane catheter attached to a silastic implant was surgically inserted into the jugular vein, with the other end securely connected to the implant, allowing it to exit through the skin on the rat’s back. Post-operative rats received intraperitoneal injections of meloxicam at a dosage of 0.1 mg/kg for pain relief. Rats were housed individually and given 7 days to recover before the start of self-administration training. During both the recovery and training phases, catheters were flushed every 24 h with sterile saline and gentamicin (0.05 mg/kg).

### 2.3. METH Self-Administration

We performed the training procedure for METH self-administration according to previously described protocols [20,22]. On the first day of training, rats were placed in their self-administration chambers, where they remained for the entire training phase and foot shock phase. Animals had free access to food and water that were available in water bottles and feeders mounted on the walls of all self-administration chambers. Naive rats were divided into two groups: METH (*n* = 18) and saline control (n = 6). We trained rats to self-administer METH (NIDA Drug Supply, Baltimore, MD, USA) (0.1 mg/kg/infusion) on a fixed ratio-1 (FR-1) with a 20 s timeout for 20 days using a pattern of three 3 h sessions per day. Control rats were subject to the same conditions as the METH rats but were self-administering saline. Rats had 5 days each week of self-administration with 2 days off on the weekend to minimize weight loss, a well-known side effect of METH use in laboratory animals. Catheter patency was monitored throughout the experiment.

### 2.4. Foot Shock Phase

During the punishment phase, rats continued METH self-administration daily (three 3 h sessions per day, separated by 30 min off intervals) under FR-1 schedule with 20 s timeout. For METH-trained rats, 50% of the reinforced lever presses also resulted in the concurrent delivery of a 0.5 s foot shock through the grid floor [20,22]. The initial foot shock intensity was set at 0.18 mA and increased by 0.06 mA daily to a final value of 0.36 mA (a total of 9 punishment days).

### 2.5. Isolation of Prefrontal Cortex, Nucleus Accumbens, Dorsal Striatum, and Midbrain and RNA Extraction

Rats were euthanized on day 30 by rapid decapitation with a guillotine 2 h after they self-administered METH in the presence of foot shock. Prefrontal cortex (PFC), nucleus accumbens (NAc), dorsal striatum (DStr), and midbrain (MBr) were isolated from the brains using precise neuroanatomical coordinates using the Atlas [23] and immediately snap-frozen on dry ice and stored at −80 °C. Qiagen RNeasy Mini kit (Qiagen, Germantown, MD, USA) was used to isolate total RNA from prefrontal cortex, nucleus accumbens, dorsal striatum, and midbrain. Isolated RNA was quantified using NanoDrop 2000 (Thermo Fisher Scientific, Waltham, MA, USA). Estimation of RNA integrity (RIN) was analyzed using Agilent Bioanalyzer 2100 (Santa Clara, CA, USA), and RNA samples with RIN 8 or above were used for RNA sequencing.

### 2.6. RNA Sequencing and Data Analysis

RNA was sent to NIH Intramural Sequencing Center (NISC, Rockville, MD, USA), and a total of 72 high-quality RNA samples (RIN > 8) were sequenced, with six samples per brain region (PFC, NAc, DStr, and MBr) for each of the three phenotypes (CT, SR, and SS). In brief, Stranded poly-A selected mRNA libraries were constructed from ~1 µg total RNA using NEBNext Poly(A) mRNA Magnetic Isolation Module (NEB #E7490, Ipswich, MA, USA) and NEBNext Ultra II Directional RNA Library Prep for Illumina with Sample Purification Beads (NEB #E7765, Ipswich, MA, USA). All samples were processed together on a Beckman i7 robot (Beckman, San Jose, CA, USA). Amplification was performed using 9 cycles of PCR. Unique dual-indexed barcode adapters were applied to each library. Libraries were pooled in an equimolar ratio for sequencing. The pooled libraries were sequenced on a 25B flow cell on a NovaSeq X Plus DNA Sequencer (Illumina, Inc., San Diego, CA, USA) to generate a minimum of 60 M 150 b paired-end reads.

RNA sequencing data analysis was performed as per previously published protocols [24,25,26,27,28] (please refer to Materials and Methods Section S1.1). In brief, raw data analyzed from RNA sequencing was analyzed using the Galaxy platform (version 24.1) [24]. Initial quality assessment of the raw sequencing reads was performed using FastQC [25], which evaluated the per-base sequence quality, GC content, N content, and sequence length distribution, and detected any overrepresented sequences or adapter contamination. The individual FastQC reports for each sample were then collected and summarized using MultiQC [26], which generated an interactive aggregate report of quality metrics across all samples and confirmed that no sample had anomalously poor read quality or other issues requiring exclusion or additional preprocessing. Adapters and low-quality sequences were removed from the reads using Trimmomatic (version 0.27) [27], which was run in paired-end mode with a custom adapter sequence provided to the ILLUMINACLIP parameter. Cleaned paired-end reads were aligned to the Rattus norvegicus reference genome using the STAR aligner [28]. The specific reference genome used was the Ensembl mRatBN7.2 assembly (Ensembl release 112), with the corresponding Ensembl gene annotation GTF file (release 112) provided to STAR for guiding splice-aware alignment. Gene-level quantification of aligned reads was carried out using FeatureCounts (version 2.0.6) to generate gene expression levels. Subsequent data analysis, including normalization and differential expression, was performed using DESeq2 for all comparisons. *p*-values and log2 fold changes were generated using the WALD test. RNA sequencing data was deposited to **NCBI GEO, accession number GSE301346**.

### 2.7. Statistical Analyses

Behavioral data were analyzed using GraphPad Prism (version 10.6.0, Boston, MA, USA) with factorial ANOVA with repeated measures. The independent variables were the rat reward types (saline, shock-sensitive, and shock-resistant), and the within-subject factor was self-administration (SA) day (training days 1–20). The dependent variable was methamphetamine intake. When a significant main effect was detected, Tukey’s post hoc multiple comparison test was performed. Statistical significance for all hypothesis tests was set at *p* < 0.05.

## 3. Results

### 3.1. Rats That Self-Administered Methamphetamine Are Separated into Compulsive (Shock-Resistant) and Non-Compulsive (Shock-Sensitive) Behavioral Phenotypes When Introduced to Foot Shocks

**Figure 1:**
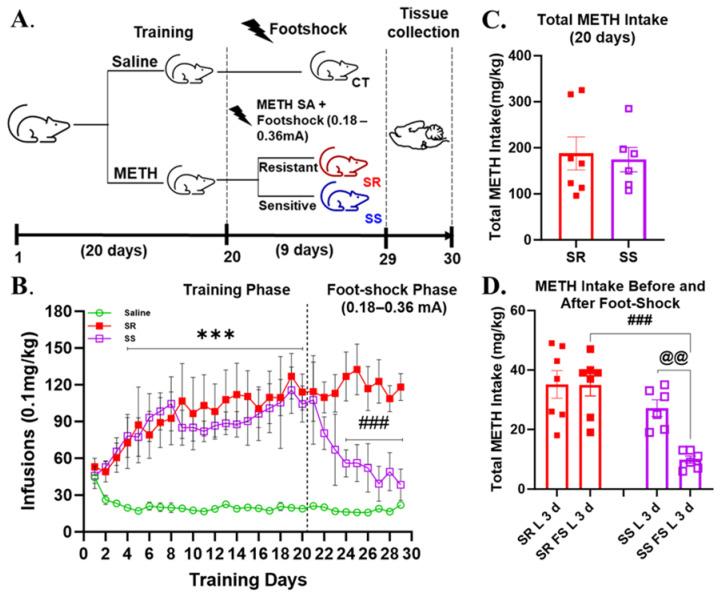
METH self-administration and contingent foot shocks result in compulsive METH taking in a subpopulation of rats. (**A**) Experimental timeline. (**B**) The figure shows a steady rise in METH infusions for the first 20 days in both SR and SS groups and a marked reduction in METH infusions in the SS rats during the 9-day foot shock phase. (**C**) The figure shows a non-significant difference in total METH intake over the first 20 days of the training phase. (**D**) The figure shows METH intake during the last 3 days of training without shocks (L 3 d) and the last 3 days of the foot shock phase (FS L 3 d). CT, saline; SR, shock-resistant; SS, shock-sensitive. Key to statistics: (1) *** *p* < 0.001, control compared with SR or SS; (2) ### *p* < 0.001, SR vs. SS comparisons; (3) @@ *p* < 0.01, comparison of drug intake during the last 3 days of METH SA vs. drug intake during the last 3 days of the foot shock phase.

Figure 1A presents the experimental timeline for this behavioral study. During the 20-day period of self-administration (SA) training, the rats that self-administered METH increased their drug intake, while the saline animals decreased their intake, and the ANOVA revealed a significant training days x group interaction [F(19, 323) = 3.742, *p* < 0.0001] (Supplementary Figure S1). On day 21, METH SA rats were subjected to foot shocks with intensities gradually increasing from 0.18 to 0.36 mA over the subsequent 9 days. Based on criteria from our previously published work, rats that decreased their METH intake by 60% or more in the presence of foot shock were classified as shock-sensitive (SS), while those that continued to self-administer METH despite the punishment were designated as shock-resistant (SR) [29,30]. After the foot shock phase, we observed that 54% of rats were classified as SR and 46% as SS (Figure 1B). Based on foot shock responses, we reanalyzed the behavioral data. A repeated-measures ANOVA of the first 20 days of SA revealed significant main effects for training days [F(4.064, 65.02) = 3.972, *p* = 0.0058] and phenotype [F(2, 16) = 8.490, *p* = 0.0031] and a significant interaction between them [F(38, 304) = 2.219, *p* = 0.0001]. A post hoc analysis indicated that both SR and SS rats significantly escalated their METH intake over the 20-day period compared to saline SA rats; however, no significant differences were observed between SR and SS rats during this phase (Figure 1B). When we analyzed the infusion data for SR and SS phenotypes during the foot shock phase, we found significant main effects for training days [F(3.371, 53.94) = 3.353, *p* = 0.0213] and phenotype [F(2, 16) = 13.63, *p* = 0.0004] and a significant interaction between training days and phenotype [F(22, 176) = 3.579, *p* < 0.0001]. SR rats self-administered significantly more METH during the foot shock phase compared to both SS rats and saline controls.

An analysis of total METH intake over the initial 20 days of SA training also revealed no significant differences between the SR and SS rats [F(6,5) = 2.146, *p* = 0.4197] (Figure 1C). When comparing METH intake during the last 3 days of SA versus the last 3 days of the foot shock phase, we observed a significant effect [F(3, 22) = 4.563, *p* = 0.0124]. Specifically, SS rats exhibited a significant reduction in total METH intake during the last 3 days of the foot shock phase relative to the final 3 days of SA training (Figure 1D), an effect not observed in SR rats. Furthermore, SR rats self-administered significantly more METH than SS rats during the last 3 days of the foot shock phase (Figure 1D).

### 3.2. RNA Sequencing Revealed Dynamic Transcriptome Reprogramming in Both Compulsive and Non-Compulsive METH-Taking Rats

**Figure 2:**
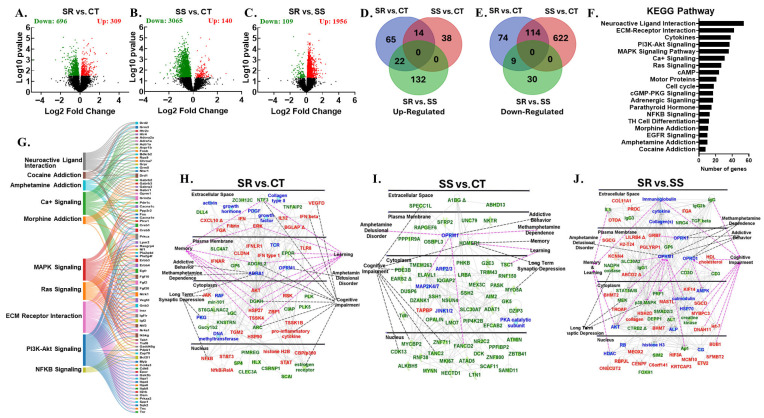
Prefrontal cortex RNA sequencing data. An analysis of raw sequencing data using log2 fold-changes and log10 *p*-values revealed many differentially expressed genes in pairwise comparisons shown as volcano plots: (**A**) SR vs. CT, (**B**) SS vs. CT, and (**C**) SR vs. SS. The Venn diagram in (**D**) illustrates common and unique up-regulated genes between 3 pairwise comparisons: SR vs. CT, SS vs. CT, and SR vs. SS. The Venn diagram in (**E**) depicts common and unique down-regulated genes between 3 pairwise comparisons: SR vs. CT, SS vs. CT, and SR vs. SS. (**F**) The KEGG analysis shows the pathway distribution of differentially expressed genes according to DAVID. (**G**) Sankey diagrams illustrate the multifunctional involvement of DEGs across multiple pathways related to substance use disorder. (**H**) Ingenuity pathway analysis identifies networks in pathways and genes significantly enriched in the SR vs. CT comparison. (**I**) Pathways and genes significantly enriched in the SS vs. CT comparison. (**J**) Pathways and genes significantly enriched in the SR vs. SS comparison. Red indicates up-regulated genes, green represents down-regulated genes, and blue represents interacting gene partners.

**Figure 3:**
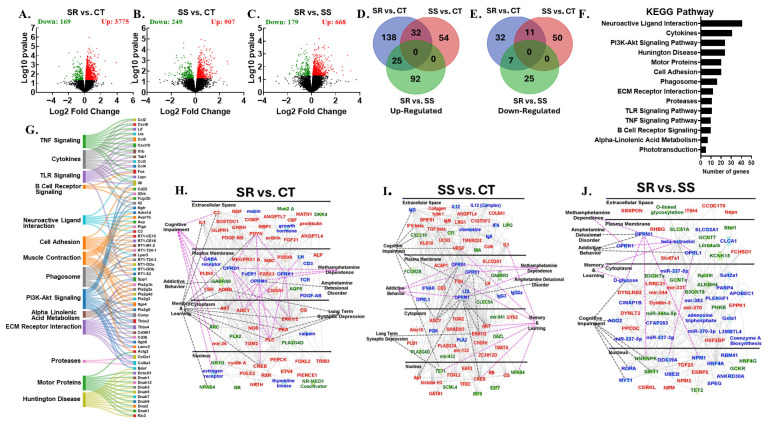
Nucleus accumbens RNA sequencing data. The analysis of raw sequencing data using log2 fold-changes and log10 *p*-values identified numerous differentially expressed genes in pairwise comparisons, presented as volcano plots: (**A**) SR vs. CT, (**B**) SS vs. CT, and (**C**) SR vs. SS. The Venn diagram in (**D**) shows the common and unique up-regulated genes across the three pairwise comparisons: SR vs. CT, SS vs. CT, and SR vs. SS. The Venn diagram in (**E**) illustrates the common and unique down-regulated genes across the three pairwise comparisons: SR vs. CT, SS vs. CT, and SR vs. SS. (**F**) The KEGG analysis displays the pathway distribution of differentially expressed genes based on DAVID. (**G**) Sankey diagrams illustrate the multifunctional involvement of DEGs across multiple pathways related to substance use disorder. The Ingenuity Pathway Analysis (IPA) identifies networks in (**H**) pathways and genes significantly enriched in the SR vs. CT comparison, (**I**) pathways and genes significantly enriched in the SS vs. CT comparison, and (**J**) pathways and genes significantly enriched in the SR vs. SS comparison. Red indicates up-regulated genes, green indicates down-regulated genes, and blue indicates interacting gene partners.

**Figure 4:**
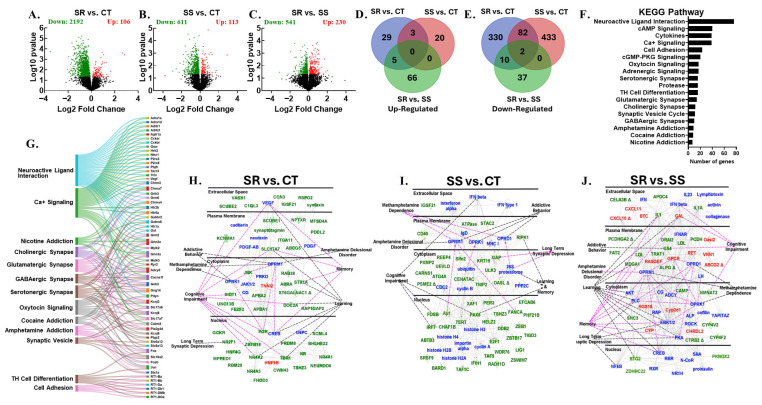
Dorsal striatum RNA sequencing data. An analysis of raw sequencing data using log2 fold-changes and log10 *p*-values revealed several differentially expressed genes in pairwise comparisons, displayed as volcano plots: (**A**) SR vs. CT, (**B**) SS vs. CT, and (**C**) SR vs. SS. The Venn diagram in (**D**) shows the common and unique up-regulated genes across the three pairwise comparisons: SR vs. CT, SS vs. CT, and SR vs. SS. The Venn diagram in (**E**) highlights common and unique down-regulated genes between the same three pairwise comparisons. (**F**) The KEGG analysis reveals the pathway distribution of differentially expressed genes according to DAVID. (**G**) Sankey diagrams illustrate the multifunctional involvement of DEGs across multiple pathways related to substance use disorder. The Ingenuity Pathway Analysis (IPA) identifies networks in (**H**) pathways and genes significantly enriched in the SR vs. CT comparison, (**I**) pathways and genes significantly enriched in the SS vs. CT comparison, and (**J**) pathways and genes significantly enriched in the SR vs. SS comparison. Red represents up-regulated genes, green represents down-regulated genes, and blue represents interacting gene partners.

**Figure 5:**
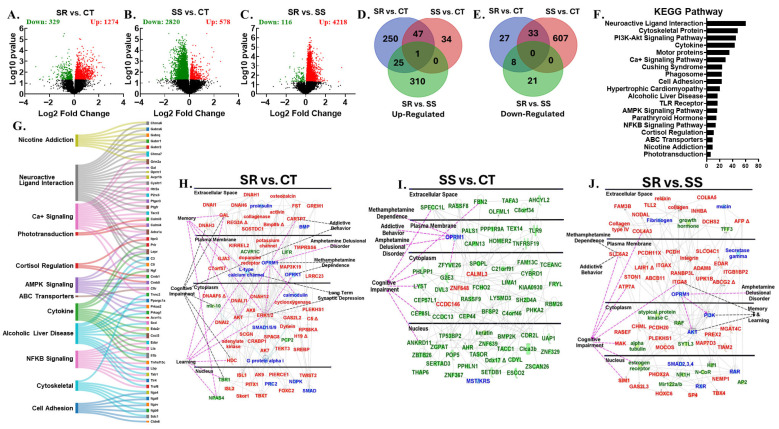
Midbrain RNA sequencing data. The analysis of raw sequencing data using log2 fold-changes and log10 *p*-values revealed multiple differentially expressed genes in pairwise comparisons, shown as volcano plots: (**A**) SR vs. CT, (**B**) SS vs. CT, and (**C**) SR vs. SS. The Venn diagram in (**D**) illustrates common and unique up-regulated genes across the three pairwise comparisons: SR vs. CT, SS vs. CT, and SR vs. SS. The Venn diagram in (**E**) displays common and unique down-regulated genes across the same three pairwise comparisons. (**F**) The KEGG analysis shows the pathway distribution of differentially expressed genes according to DAVID. (**G**) Sankey diagrams illustrate the multifunctional involvement of DEGs across multiple pathways related to substance use disorder. The Ingenuity Pathway Analysis (IPA) identifies networks in (**H**) pathways and genes significantly enriched in the SR vs. CT comparison, (**I**) pathways and genes significantly enriched in the SS vs. CT comparison, and (**J**) pathways and genes significantly enriched in the SR vs. SS comparison. Red represents up-regulated genes, green represents down-regulated genes, and blue represents interacting gene partners.

**Figure 6:**
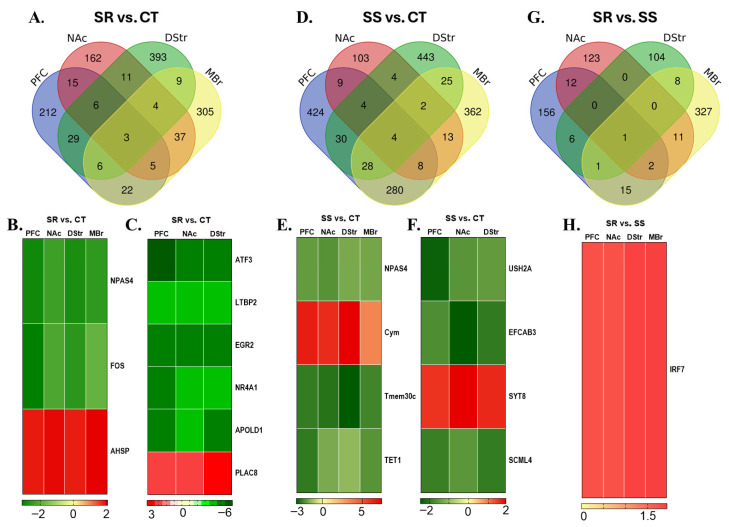
Combination analysis of RNA sequencing data. (**A**) The Venn diagram reveals unique and shared genes in the PFC, NAc, DStr, and MBr for the SR vs. CT comparison. (**B**) shows common DEGs among PFC, NAc, DStr, and MBr for the SR vs. CT comparison, and (**C**) shows 6 differentially expressed genes in the PFC, NAc, and Dstr in the SR vs. CT comparison. (**D**) The Venn diagram illustrates the common and unique genes across the PFC, NAc, DStr, and MBr in the SS vs. CT comparison. (**E**) Hierarchical clustering of 4 differentially expressed genes in the PFC, NAc, DStr and MBr in the SR vs. CT comparison; (**F**) 4 differentially expressed genes in the PFC, NAc, and DStr in the SS vs. CT comparison. (**G**) The Venn diagram shows the common and unique genes across the PFC, NAc, DStr, and midbrain in the SR vs. SS comparison. (**H**) Hierarchical clustering of 1 differentially expressed gene in the PFC, NAc, DStr, and MBr in the SR vs. SS comparison. The color scale indicates the relative expression levels, with red representing up-regulation and green indicating down-regulation.

We hypothesized that the differences between SR and SS phenotypes are linked to altered neural activity in brain reward circuits. The dopaminergic mesocorticolimbic and nigrostriatal systems, including the PFC, NAc, DStr, and midbrain, are key regions involved in reward, learning, decision-making, and habit formation, all contributing to METH use disorder (MUD) [12,21,29,30,31,32,33]. This led us to study the global transcriptional changes associated with METH use in PCF, NAc, DStr, and MBr. We conducted RNA sequencing to identify potential gene networks underlying compulsive and non-compulsive behaviors in response to punishment (Figure 2, Figure 3, Figure 4, Figure 5 and Figure 6). Using DESeq2, we performed three pairwise comparisons (SR vs. CT, SS vs. CT, and SS vs. SR) for each brain region we studied. Sequencing revealed identified dynamic transcriptional reprogramming among compulsive and non-compulsive rats, represented as volcano plots, as illustrated in Figure 2A–C, Figure 3A–C, Figure 4A–C and Figure 5A–C. To focus on biologically meaningful changes, we applied a dual filtering criterion, requiring both a fold change ≥ 1.5 and a *p*-value < 0.05. This more stringent cutoff was used to identify genes for downstream analyses and network mapping. Differentially expressed genes (DEGs) meeting these criteria were then compared between SR and SS phenotypes, and the unique and shared DEGs were visualized using Venn diagrams (Figure 2D,E, Figure 3D,E, Figure 4D,E and Figure 5D,E). Expression patterns of DEGs are illustrated using hierarchical clustering (Supplementary Figure S2). The Database for Annotation, Visualization and Integrated Discovery (DAVID) was used for functional annotation and clustering of DEGs, and the results are presented as KEGG pathway analyses (Figure 2F, Figure 3F, Figure 4F and Figure 5F). Sankey diagrams illustrated the multifunctional involvement of DEGs across multiple pathways related to substance use disorder (Figure 2G, Figure 3G, Figure 4G and Figure 5G). Finally, Qiagen’s Ingenuity Pathway Analysis (IPA) was used to identify gene networks for SR vs. CT (Figure 2H, Figure 3H, Figure 4H and Figure 5H), SS vs. CT (Figure 2I, Figure 3I, Figure 4I and Figure 5I), and SR vs. SS comparisons (Figure 2J, Figure 3J, Figure 4J and Figure 5J). What follows is the detailed RNA sequencing results for PFC, NAc, DStr, and MBr. Lastly, we also identified DEGs which were common among all four brain regions under study.

### 3.3. Differentially Expressed Genes in Prefrontal Cortex

RNA sequencing revealed widespread transcriptional changes in the PFC of shock-resistant (SR) (309 up- and 696 down-regulated genes) and shock-sensitive (SS) (140 up- and 3065 down-regulated genes) rats when compared to CT (Figure 2A,B) and in SR (1956 up- and 109 down-regulated genes) compared to SS (Figure 2C). We applied a stringent cutoff of ≥1.5-fold change and a *p*-value < 0.05 to identify differentially expressed genes. Using these criteria, we found unique gene expression changes in the following comparisons: SR vs. CT (65 up-regulated and 74 down-regulated genes), SS vs. CT (38 up-regulated and 622 down-regulated genes), and SR vs. SS (132 up-regulated and 30 down-regulated genes) (Figure 2D,E). We identified 22 up-regulated and 9 down-regulated shared genes among the SR vs. CT and SR vs. SS comparisons (Figure 2D,E). These shared DEGs may be more relevant to compulsive METH intake as they were altered in compulsive SR rats compared to the CT and SS groups. Furthermore, the SR vs. CT and SS vs. CT comparisons also revealed shared genes, namely 14 up-regulated and 114 down-regulated genes (Figure 2D,E). Hierarchical clustering of all DEGs across all comparisons revealed distinct expression patterns (Supplementary Figure S2A): the genes mostly remained unchanged in SR vs. CT but were decreased in SS vs. CT. This might explain why the SR vs. SS comparison showed a higher number of up-regulated genes. The KEGG pathway analysis performed using DAVID grouped DEGs into functional categories, which include neuroactive ligand–receptor interaction (54 genes), morphine addiction (11 genes), amphetamine addiction (10 genes), and cocaine addiction (8 genes) (Figure 2F). Sankey diagrams (Figure 2G) illustrated the multifunctional involvement of DEGs across multiple pathways related to substance use disorder. Ingenuity Pathway Analysis (IPA) identified biological gene networks for DEGs, further demonstrating their role in METH dependance, amphetamine delusional disorder, addictive behaviors, and cognitive impairment. IPA also identified key DEGs with functional relevance to METH use disorder, including *Erk*, *Nfkb*, *Akt*, and *Vegfd*, which were up-regulated, and *Ntf3*, *Arc*, *Adgrl2*, and *Dgkh*, which were down-regulated in SR vs. CT (Figure 2H). Genes like *Homer1*, *Tanc2*, *Fancd2*, *Cdk13*, and *Elavl1* were down-regulated in SS vs. CT (Figure 2I). The SR vs. SS comparison displayed a higher mRNA level for *Grb7*, *Sgcd*, *Mtbpc3*, and *Kcnn4*, whereas *Slc30a2*, *Prf1*, *Stat5a*, and *Il1* expression decreased (Figure 2J).

### 3.4. Differentially Expressed Genes in Nucleus Accumbens

An analysis of raw sequencing data for NAc revealed DEGs among three pairwise comparisons, SR vs. CT (3775 up- and 169 down-regulated, Figure 3A), SS vs. CT (907 up- and 249 down-regulated, Figure 3B), and SR vs. SS (668 up- and 179 down-regulated, Figure 3C). Further analysis of sequencing data was performed with a stringent cutoff of ≥1.5-fold change and a *p*-value < 0.05 to identify biological meaningful DEGs. This revealed 170 unique (138 up and 32 down) DEGs in SR vs. CT; 104 unique (54 up and 50 down) DEGs in SS vs. CT; and 117 unique (92 up and 25 down) DEGs in SR vs. SS (Figure 3D,E). The Venn diagram further revealed 32 up-regulated and 11 down-regulated shared genes among the SR vs. CT and SS vs. CT comparisons (Figure 3D,E). Compulsive METH-taking (SR) rats presented common genes (25 up- and 7 down- regulated) when SR vs. CT and SR vs. SS were compared together (Figure 3D,E). The expression of all the DEGs across the three pairwise comparisons is presented as hierarchical clustering (Supplementary Figure S2B), which revealed distinct expression patterns. DAVID was used to generate functional annotation and clustering for these 1525 DEGs. Functional gene clusters and KEGG pathways grouped the altered genes into major categories, which include neuroactive ligand–receptor interaction (41 genes), cytokine signaling (31 genes), and Huntington’s disease (24 genes) (Figure 3F), and Sankey diagrams further illustrated how specific DEGs were involved in multiple KEGG pathways (Figure 3G). IPA further identified the key gene networks underlying behavioral and neurobiological alterations. Of significant relevance, *C3*, *Il1*, *Ngf*, *Pdyn*, *Fgf21*, *Fsh*, *Erk*, *Pka*, *Plc*, and *Creb* were up-regulated, while *Gabra6*, *Arc*, *Nr1i3*, and *Npas4* were down-regulated in the SR vs. CT comparison (Figure 3H). Additionally, *Vegf*, *Il1*, *Akt*, *Erk*, *Adcy*, *Ap1*, and *Creb* expression levels were increased, while *Gabrr2*, *Fcgr2b*, *Tet1*, and *Npas4* levels were decreased in the SS vs. CT comparison (Figure 3I). For the SR vs. SS comparison, *Slc47a1*, *Eppk1*, *Tcf23*, *Nmp*, and *Nepn* mRNA levels were increased, whereas *Kcnk15*, *Hnrnpk*, *Sirt1*, and *Tet2* showed reduced expression (Figure 3J).

### 3.5. Differentially Expressed Genes in Dorsal Striatum

In the DStr, we observed large-scale transcriptional reprogramming, which was associated with the amount of METH that the rats self-administered during the foot shock phase. When the SR rats were compared with the CT rats, it was seen that 106 genes were up-regulated, while 2192 genes were down-regulated (Figure 4A). The SS vs. CT comparison revealed that the mRNA levels of 113 genes were elevated, whereas those of 611 genes were reduced (Figure 4B). When we compared SR vs. SS, the expression of 230 genes was increased, while the expression of 541 genes was found to be decreased (Figure 4C). Using a more stringent cutoff of ≥1.5-fold change and a *p*-value < 0.05, the Venn diagram revealed uniquely differentially regulated genes as follows: 29 up-regulated and 330 down-regulated genes in SR vs. CT; 20 up-regulated and 433 down-regulated genes in SS vs. CT; and 66 up-regulated and 37 down-regulated genes in SR vs. SS (Figure 4D,E). We observed the least number of shared genes (5 increased and 10 decreased) when SR vs. CT and SR vs. SS were compared together (Figure 4D,E). The comparisons of SR vs. CT and SS vs. CT revealed 3 up- and 82 down- regulated genes (Figure 4D,E). Moreover, NKX1-2 and BTG2 showed consistent down-regulation across all three pairwise comparisons (Figure 4D,E). Hierarchical clustering revealed distinct expression patterns of all DEGs across the three pairwise comparisons (Supplementary Figure S2C). Furthermore, the functional annotation of DEGs highlighted several enriched KEGG pathways, which include neuroactive ligand–receptor interaction (76 genes), oxytocin signaling (17 genes), Ca+ signaling (39 genes), serotonergic synapse (16 genes), Glutamatergic synapse (14 genes), cholinergic synapse (12 genes), GABAergic synapse (10 genes), and genes involved in amphetamine (9 genes), cocaine (8 genes), and nicotine (7 genes) addictions (Figure 4F). We also generated a Sanky diagram to identify the common DEGs across multiple biological pathways, which is illustrated in Figure 4G. The down-regulated genes in the SR vs. CT comparison included *Kcnma1*, *Sidt1*, *Pgr*, *Tbr1*, *Nr4a2*, *Nr4a3*, *Nr2f1*, *Stx1a*, *Doc2a*, and *Pgr*, and those in the SS vs. CT comparison included *Ripk1*, *Fosb*, *Ap1*, *Tet1*, *Pax6*, and *Fanca*. In SR vs. SS comparison genes which were increased included *Gal*, *Oasl2*, *Btc*, and *Gpcr*, and those that were decreased included *Il1*, *Mdga1*, *Nmnat2*, *Shc3*, and *Btg2*. All of these DEGs were found to be associated with METH dependance, amphetamine delusional disorder, addictive behaviors, long-term synaptic depression, and cognitive impairment (Figure 4H–J).

### 3.6. Differentially Expressed Genes in Midbrain

Finally, we identified global transcriptional changes in the MBr of METH self-administered rats. Specifically, the SR vs. CT comparison revealed 1274 up- and 329 down-regulated genes (Figure 5A), while in the SS vs. CT expression, 578 and 2820 genes were increased and decreased, respectively (Figure 5B), with the SR vs. SS comparison showing higher mRNA levels for 4218 genes and lower levels for 116 genes (Figure 5C). Furthermore, using a stringent cutoff of ≥1.5-fold change and a *p*-value < 0.05, we identified unique genes in all three pairwise comparisons: SR vs. CT (250 up-regulated and 27 down-regulated), SS vs. CT (34 up-regulated and 607 down-regulated), and SR vs. SS (310 up-regulated and 21 down-regulated) (Figure 5D,E). Only *Isl2* was found to be up-regulated across all three pairwise comparisons (Figure 5D). The Venn diagram also revealed shared DEGs among SR vs. CT and SS vs. CT (47 up-regulated and 33 down-regulated genes) and SR vs. CT and SR vs. SS (25 up-regulated and 8 down-regulated genes) when compared (Figure 5D,E). The pattern of expression of these DEGs is illustrated as a hierarchical cluster (Supplementary Figure S2D). Like other brain regions, the KEGG pathway analysis revealed 60 genes involved in neuroactive ligand interaction, along with genes involved in alcoholic liver disease (17 genes), cortisol regulation (11 genes), and nicotine addiction (9 genes) (Figure 5F). Functional diversity was visualized with Sankey diagrams (Figure 5G). An IPA network analysis for MBr also showed an association between METH dependance, amphetamine delusional disorder, addictive behaviors, long-term synaptic depression, and cognitive impairment and the identified DEGs in the three pairwise comparisons, namely SR vs. CT (*Gal, Cartpt, Akt, Erk, Hdc, Isl1, Isl2*, and dopamine receptors were up-regulated, and *Tbr1* and *Npas4* were down-regulated), SS vs. CT (*Calml3* and *Znf648* were up-regulated, while *Fbn2, Zfyve26, Lyst, Cep85l*, and *Ankrd11* were down-regulated), and SR vs. SS (*Col4a3, Slc6a2, Atp7a*, and *Itga8* were up-regulated) (Figure 5H–J).

### 3.7. Shared Differentially Expressed Genes Across Multiple Brain Regions

We also sought to identify DEGs that were common in the prefrontal cortex (PFC), nucleus accumbens (NAc), dorsal striatum (DStr), and midbrain (MBr). We thought that identifying the shared DEGs across multiple brain regions is of utmost importance because this might hold the key to finding better therapeutic targets against METH use disorder (MUD). In this quest, we preformed a pairwise comparison across four brain regions for SR vs. CT, SS vs. CT, and SR vs. SS, and the results are presented as a Venn diagram (Figure 6A,D,G), as heatmaps (Figure 6B,C,E,F,H), and in Supplementary Table S1. We found DEGs which were shared across PFC, NAc, DStr, and MBr (Figure 6B,E,H). In the SR vs. CT comparison, *Npas4* and *Fos* were down-regulated, while *Ahsp* was up-regulated across all four regions (Figure 6B). The mRNA expression of *Tet1*, *Npas4*, and *Tmem30c* was decreased, while that of *Cym* (Chymosin) increased in the non-compulsive rats (SS vs. CT) and the controls in all brain regions studied (Figure 6E). Interestingly, the SR vs. SS comparison showed *Irf7* as a common gene with increased expression in PFC, NAc, DStr, and MBr (Figure 6H).

We also searched for the common gene in the PFC, NAc, and DStr because the brain structure receives neuronal projections from the midbrain. Moreover, mesocorticolimbic and nigrostriatal projections from the MBr is central to the brain’s reward system and plays an important role in the rewarding effects of drug abuse and in the development of substance use disorders. The compulsive (SR) rats displayed increased expression of *Plac8*, whereas *Nr4a1*, *Atf3*, *Ltbp2*, *Egr*2, and *Apold1* were decreased in the PFC, NAc, and DStr of compulsive rats when compared to CT (Figure 6C). The mRNA level of *Syt8* was found to be higher, while lower levels were seen for *Ush2a*, *Efcab3*, and *Scml4* in the PFC, NAc, and DStr of non-compulsive (SS) rats compared to the control animals (Figure 6F). Interestingly, no common DEG was observed in the PFC, NAc, and DStr in the SR vs. SS comparison (Figure 6G). We also found shared DEGs like (1) *Cdhr3, Mfge8, Plin5, Tssk4,* and *Lilrb3* in the PFC, NAc, and MBr; (2) *Abra, Nags, Dll4, Cym, Ca3*, and *Hemgn* in the PFC, DStr, and MBr; and (3) *Sult1a1, Ldlrad2, Dpep2*, and *Pla2g4d* in the NAc, DStr, and MBr of SR rats compared to the CT rats (Supplementary Figure S3A–C). Moreover, SS rats showed shared DEGs, namely (1) *Hspa1a, Clca1, Trim69, Slc27a6, Mfge8, Mir-186, Lilrb3*, and *Ago3* in the PFC, NAc, and MBr; (2) *Nbeal1, Grifin, Gan, Col4a3, Nr5a1, Zgrf1, Irf7, Npm2, Xiap, Ctrb2, Gk5, Hipk3, Zc3h12c, Serpinf2, Prss30, Ctnna3, Dll4, Creb5, Impg2, Piga, Tmem245, Prr5l, St6gal2, Kcnh5, Igsf9b, Frmd7, Uevld*, and *Atp10b* in the PFC, DStr, and MBr; and (3) *Znf804b* and *Nepn* in the NAc, DStr, and MBr when compared to the CT rats (Supplementary Figure S3D–F). Finally, (1) *Abcg2* and *Ush2a* were common to the PFC, NAc, and MBr, while only *H2-t24* was shared across the PFC, DStr, and MBr in the SR rats compared to the SS rats (Supplementary Figure S3G,H).

## 4. Discussion

The present study investigated the effects of foot shock on METH self-administration and, using RNA sequencing, we measured the global transcriptional changes in four different brain regions of two drug self-administering phenotypes. Over the course of the 20-day training phase, all METH SA rats escalated their drug intake. During the 9-day foot shock phase, a clear phenotypic division emerged, separating rats into compulsive (shock-resistant) and non-compulsive (shock-sensitive) groups. This result aligns with previously published studies, where researchers also reported two distinct phenotypes when rats were subjected to foot shock punishment [20,22,29,30,34]. Evidence suggests that the observed behaviors are linked to persistent neuroadaptations and transcriptional reprogramming within key brain regions of the reward circuitry, including the PFC, NAc, DStr, and MBr [13,14,15,16], accompanied by dysregulation of dopaminergic neuron signaling. METH-induced dopamine release from the midbrain reinforces drug-seeking behavior, while control over behavior shifts from the PFC to habit-related regions like the DStr. This shift weakens executive control and promotes compulsive use and relapse [13,14,15,16]. Despite these insights, the distinct region-specific effects of METH-induced neuroadaptations remain poorly understood and pose a significant barrier in the development of effective treatments against MUD. Recent multi-omics studies have begun identifying novel therapeutic targets, offering promising avenues for region-specific interventions [35,36,37,38]. This has led us to employ high-throughput sequencing as a discovery-based approach to identify critical novel molecular networks involved in methamphetamine use disorder, which may ultimately enable the development of more effective therapeutic interventions. RNA sequencing revealed large transcriptional reprogramming in the PFC, NAc, DStr, and MBr of compulsive as well as non-compulsive rats (Figure 2, Figure 3, Figure 4 and Figure 5).

### 4.1. Molecular Mechanisms Associated with Compulsive METH Intake

Observed behavioral differences were also supported by RNA-sequencing data, which identified molecular networks and genes with altered mRNA expression, specifically in compulsive rats (see Figure 2H, Figure 3H, Figure 4H and Figure 5H). Notably, rats that showed resistance to foot shocks displayed distinct gene network alterations depending on the brain region. Of particular interest, diacylglycerol kinase eta (*Dgkh*), which was down-regulated in the PFC, has been linked to bipolar disorder [39,40], unipolar depression, ADHD [41], and panic disorder [42]. Altered expression of *Nfkb*, *Erk*, and *Ntf3* in the PFC has been implicated in neuronal excitability, synaptic transmission, and plasticity and may affect the neuro-molecular mechanisms of the brain’s reward system in compulsive rats, potentially driving increased METH intake [43,44,45,46,47]. Changes in *Pdyn*, *Ngf*, and *Creb* mRNA expression in the NAC are also of significant interest. *Ngf* and *Pdyn* are integral to the reward circuitry and have been implicated in the development of substance use disorders [18,21,48,49]. Our current observations of increased *Pdyn*, *Ngf*, and *Creb* mRNA levels in compulsive rats align with previous studies, which showed increased *Pdyn* expression in the NAc and DStr [21,48] and elevated *Creb* levels in the DStr following METH exposure [18,19]. Furthermore, *Ngf* was also found to be increased in the amygdala after morphine self-administration and foot shock exposure [49]. *Kcnma1*, a calcium-gated potassium channel involved in neuronal excitability and synaptic plasticity [50,51], was found to be down-regulated in the DStr of compulsive rats. This decrease is of particular interest as previous studies reported increased *Kcnma1* expression in non-addicted METH-exposed rats [20]. Similarly, reduced striatal expression of *Nr4a2* has been documented in rats chronically administered METH [52]. Transcription factors, *Nr4a2*, and *Nr4a3* are critical for dopamine signaling and neuronal survival [53]. Consistent with earlier findings, the expression of dopamine receptors [18], *Cartpt* [54,55], and *Slc6a*2 [56,57] increased in the midbrain. These genes are implicated in addiction to psychostimulants, nociception, neuroprotection, reward, and reinforcement [18,54,55,56,57]. *Arc*, a gene critical for memory consolidation and neuronal plasticity [58,59], is known to regulate neuroadaptations underlying METH-induced behavioral sensitization [60] and was found to be decreased in both the PFC and NAc in the present study.

Together, these genes form an interconnected molecular network that regulates key processes such as dopamine signaling, synaptic plasticity, neuronal excitability, and stress response. Dysregulation of this network across brain regions involved in reward and reinforcement may promote neuroadaptations that weaken inhibitory control, enhancing compulsive METH intake despite adverse consequences. Overall, our observations support the idea that simultaneous examination of molecular alterations across different brain regions involved in the reward circuitry is essential for developing effective therapeutic targets against METH use disorder (MUD).

### 4.2. Non-Compulsive Behavior and Differential Gene Expression

To date, there are no FDA-approved medications for MUD. We believe that studying non-compulsive animals is important because they reduce their METH intake in the presence of foot shock. The genes and the molecular networks they reside in could potentially serve as targets for identifying successful therapeutic interventions, ultimately helping to develop and improve treatment options for addiction. We identified specific genes across multiple brain regions in non-compulsive rats that are associated with learning and memory, addictive behavior, cognitive impairment, methamphetamine dependence, amphetamine-induced delusional disorder, and long-term synaptic depression. In the PFC, decreased expression was observed for *Homer1*, which plays a crucial role in the postsynaptic density of excitatory synapses [61], and *Tanc2*, which helps maintain synaptic structure and function and has been implicated in psychiatric disorders [62]. In terms of substance use disorders, repeated cocaine exposure has been found to reduce *Homer1* expression in the NAc, and this reduction is associated with the development of cocaine-induced behavioral sensitization [63,64]. *Elavl1*, decreased in the PFC, is an RNA-binding protein (RBP) that increases mRNA stability [65], potentially acting as a regulator of gene transcription and cellular responses related to MUD. The trophic factor, *Vegfa*, identified in this study, has also been reported to increase in the rat nucleus accumbens after four weeks of cocaine administration [66]. There is evidence implicating *Adcy* in the manifestation of opioid addiction [67,68]. Similarly, *Gabrr2*, which encodes the GABA-A receptor subunit rho2, has been associated with alcohol dependence [69]. The transcription factor *Ap1*, which was increased in the NAc and decreased in the DStr, plays a significant role in METH abuse by mediating long-term neuroadaptations in response to chronic drug exposure [70,71,72]. Altered expression of the transcription factor *Pax6* in the dorsal striatum (DStr) has been linked to cognitive and behavioral impairments observed in neurodevelopmental disorders, including WAGR syndrome and autism [73,74,75]. In terms of substance abuse, consistent with our observations, rats self-administering cocaine also displayed decreased *Pax6* expression in the PFC [76]. *Ripk1* has been implicated in METH-induced neuroinflammation and neurotoxicity [77]. Of significant interest, the expression of *Ankrd11*, *Lyst*, and *Zfyve26* was decreased in the MBr of shock-sensitive (SS) rats. Ankyrin repeat domain 11 (*Ankrd11*) is down-regulated in METH-treated cortical neurons [78] and is known to be associated with intellectual disability [79,80]. Moreover, altered expression of lysosomal trafficking regulator (*Lyst*) [81,82] and spastizin (*Zfyve26*) [83,84] leads to neuronal degeneration and neurodegenerative diseases. This insight emphasizes that these genes form a complex molecular network that underlies the vulnerability and resilience to METH use, offering promising targets for therapeutic intervention. From a clinical perspective, understanding how these genes contribute to resilience against compulsive drug use can guide the development of novel intervention, such as gene-based therapies or pharmacological agents, that enhance cognitive control, reduce neurotoxicity, and restore neural function.

### 4.3. Potential Therapeutic Approaches for Methamphetamine Use Disorder

As previously noted, distinct but interconnected brain regions play a crucial role in the development and maintenance of substance use disorders (SUDs). Identifying genes and molecular networks that show consistent alterations across multiple brain regions is key to advancing effective treatments for MUD. In the present study, the compulsive METH-taking phenotype showed a significant increase in *Ahsp* and a decrease in *Fos*, whereas non-compulsive rats showed decreased expression of *Tet1* across the PFC, NAc, DStr, and MBr, which is of considerable interest.

Alpha-hemoglobin stabilizing protein (*Ahsp*) [85,86] and *Fos* [87,88] are known to modulate the redox potential of cells under pathological conditions by preventing oxidative stress. They may play important roles in mitigating METH-induced oxidative stress and neuroinflammation [2,89]. Ten-eleven translocation methylcytosine dioxygenase 1 (*Tet1*) has also been implicated in reward and addiction mechanisms [90,91]. Specifically, Feng et al., 2015, demonstrated that reduced expression of *Tet1* in the NAc, enhances cocaine-induced conditioned place preference, while *Tet1* overexpression in this region can reverse addiction-related behaviors [91]. Furthermore, acute METH administration increases *Tet1* binding at the corticotropin-releasing hormone (Crh) promoter, regulating its expression [90].

We also identified common differentially expressed genes in the PFC, NAc, and DStr, because these regions receive neuronal projections from the MBr and are deeply involved in reward and addiction [13,14]. We found that the expression of *Atf3*, *Egr2*, and *Nr4a1* decreased, while that of *Plac8* increased in compulsive rats. As previously noted, METH intake induces neurotoxic effects through oxidative stress and neuroinflammation [2,89], and *Plac8* [92,93] and *Atf3* [94,95] are known modulators of inflammatory responses in the brain. Furthermore, transcription factors *Nr4a1* and *Egr2* have been reported to play important roles in mediating the neurobiology and behavioral consequences of substance abuse, including METH [52,96,97,98]. *Ush2a*, which was decreased in non-compulsive rats, was found to be associated with alcohol dependence [99,100]. Defects in *Efcab3* have been linked to structural anomalies across multiple brain regions [101]. Additionally, Synaptotagmin 8 (*Syt8*), which plays a role in neurotransmission and hormone secretion [102], was increased in the non-compulsive animals.

The clinical implications of these results are significant. Our findings reveal key gene expression changes across multiple brain regions that distinguish compulsive from non-compulsive METH intake. Notably, alterations in *Tet1*, *Fos*, *Ahsp*, *Atf3*, *Nr4a1*, and *Egr2* suggest shared molecular mechanisms involving oxidative stress, neuroinflammation, and reward processing. The consistent down-regulation of *Tet1* across brain regions in non-compulsive rats underlines its potential as a therapeutic target for MUD. Additionally, the differential expression of *Plac8*, *Syt8*, and *Ush2a* could be significant in the development of future treatment strategies. These findings highlight the importance of multi-region transcriptomic analyses in identifying novel molecular targets for effective interventions in MUD.

## 5. Conclusions

In conclusion, using DSM-V criteria, we identified a subset of rats that persistently self-administered methamphetamine (METH) despite aversive consequences. Through RNA sequencing, we uncovered distinct gene expression profiles differentiating compulsive from non-compulsive phenotypes, highlighting brain region-specific molecular networks in the PFC, NAc, DStr, and MBr. Notably, compulsive rats exhibited up-regulation of *Ahsp* and down-regulation of *Fos*, whereas non-compulsive rats showed increased *Cym* expression and reduced levels of *Tet1* and *Tmem30c* across these key brain areas. Furthermore, we identified a set of common genes, including *Atf3, Egr2, Nr4a1, Plac8, Ush2a, Efcab3*, and *Syt8*, that displayed consistent expression alterations in brain regions receiving midbrain projections, underscoring their potential as molecular markers of METH addiction vulnerability. Our findings carry important translational implications as they pinpoint specific molecular targets that could be explored to develop novel pharmacotherapies against MUD.

Our findings provide critical and novel insights into the molecular and behavioral consequences of prolonged METH exposure. A limitation of our study is that it was conducted exclusively using male rats. Future analyses need to integrate data from both male and female subjects to comprehensively evaluate potential sex differences in the response to METH exposure. This approach will strengthen the translational relevance of our findings and better inform the development of sex-specific interventions for MUD.

## Data Availability

All data needed to evaluate the conclusions in the paper are present in the paper and/or the supplementary materials. RNA sequencing data was submitted to NCBI GEO #GSE301346, and processed files will be made available to end-users upon formal request to the corresponding author.

## Supplementary Materials

The following supporting information can be downloaded at: https://www.mdpi.com/article/10.3390/cells14181472/s1, Figure S1: Training phase infusions of METH taking rats showing a steady significant rise in.METH infu-sions for the first 20 days; Figure S2: Heatmap analysis showing hierarchical clustering of all differential expressed genes (DEGs) across the SR vs CT; SS vs CT; and SR vs SS comparisons in the (A) prefrontal cortex, (B) nucleus accumbens, (C) dorsal striatum, and (D) midbrain. The color scale indicates the relative expres-sion levels, with red representing up-regulation and green indicating down-regulation; Figure S3: Heatmap analysis showing hierarchical clustering of differential expressed genes (DEGs) found to be shared across multiple brain regions. In the SR vs CT comparison, (A) 6 differentially expressed genes were found in the prefrontal cortex (PFC), nucleus accumbens (NAc), and midbrain (MBr); (B) 6 differentially expressed genes were found in the PFC, DStr and MBr; and (C) 4 differentially expressed genes were found in the NAc, DStr, and MBr. In the SS vs CT comparison, (D) 8 differentially expressed genes were found in the PFC, DStr, and MBr; (E) 28 differentially expressed genes were found in the PFC, DStr and MBr; and(F) 2 differentially expressed genes were found in the NAc, DStr, and MBr. In the SR vs SS comparison, (G) 2 differ-entially expressed genes were found in the PFC, NAc and MBr; and (H)1 differentially expressed genes was found in the PFC, DStr, and MBr. The color scale indicates the relative expression lev-els, with red representing up-regulation and green indicating down-regulation; Table S1. List of differential expressed genes (DEGs) shared across multiple brain regions, (A) SR vs CT (B) SS vs CT (C) SR vs SS. Green color indicate “Downregulated genes”, whereas red color indicate “Upregulated genes”.

## Abbreviations

The following abbreviations are used in this manuscript:

MUD	Methamphetamine Use Disorder
METH	Methamphetamine
SR	Shock-Resistant
SS	Shock-Sensitive
CT	Control
PFC	Prefrontal Cortex
NAc	Nucleus Accumbens
DStr	Dorsal Striatum
MBr	Midbrain
