# Supplementary material for: RNA Sequencing Identified Differentially Expressed Genes in the Mesocorticolimbic and Nigrostriatal Systems of Compulsive METH-Taking Rats
Source: Cells. 2025 Sep 20;14(18):1472. doi: 10.3390/cells14181472 (PMC12468296; doi:10.3390/cells14181472)
Supplement: Supplementary file 1 [file cells-14-01472-s001.zip › cells-3806276-supplementary.pdf]

Supplementary File

RNA sequencing identified differentially expressed genes in the mesocorticolimbic and nigrostriatal system of compulsive METH taking rats.

Nasser Adjei [1], Bruce Ladenheim [1], Michael T. McCoy [1], Vikrant Palande [2], Jean Lud Cadet [1] and Atul P. Daiwile [1*]

[1] Molecular Neuropsychiatry Research Branch, NIDA Intramural Research Program, Baltimore, MD 21224, United States of America.
[2] Department of Pathology, Johns Hopkins University, Baltimore, Maryland, United States of America.
* Correspondence: atul.daiwile@nih.gov ; Tel.: (667) 312-5079

1. Materials and Methods

S1.1. RNA Sequencing Data Analysis

Raw data analyzed from RNA sequencing was analyzed using the Galaxy platform [24]. All samples consisted of Illumina paired-end reads. To ensure reproducibility and efficient batch processing, raw FASTQ files were organized into Galaxy dataset collections. Forward and reverse reads were automatically paired and assigned sample identifiers using Galaxy's "Apply Rules" tool within the workflow.

Initial quality assessment of the raw sequencing reads was performed using FastQC [25] which evaluated per-base sequence quality, GC content, N content, sequence length distribution, and detected any overrepresented sequences or adapter contamination. The individual FastQC reports for each sample were then collected and summarized using MultiQC [26] which generated an interactive aggregate report of quality metrics across all samples and confirmed that no sample had anomalously poor read quality or other issues requiring exclusion or additional preprocessing.

Adapters and low-quality sequences were removed from the reads using Trimmomatic [27], which was run in paired-end mode with a custom adapter sequence provided to the ILLUMINACLIP parameter. Along with clipping adapters, a sliding window approach was applied to trim low-quality bases (Phred score <20) from the reads ends. Reads shorter than minimum lengths (after trimming) were discarded to avoid spurious alignments. This trimming step produced a set of cleaned paired reads for each sample. Corresponding orphaned reads (if any mate in a pair fell below length thresholds) handled according to Trimmomatic's default behavior. The effectiveness of adapter trimming and quality filtering was verified by rerunning FastQC/MultiQC on the trimmed reads, confirming the removal of adapter-derived sequences and improvement in overall read quality where necessary.

Cleaned paired-end reads were aligned to the Rattus norvegicus reference genome using the STAR aligner [28]. The specific reference genome used was the Ensembl mRatBN7.2 assembly (Ensembl release 112), with the corresponding Ensembl gene annotation GTF file (release 112) provided to STAR for guiding splice-aware alignment. STAR was run with default parameters for single-pass alignment, leveraging the gene annotation to improve the accuracy of exon–intron junction mapping. All alignments were performed in genome coordinate mode, producing sorted BAM files for each sample. Alignment quality was evaluated via STAR's alignment statistics (e.g., mapping percentage, multi-mapping rates), which were collated in the MultiQC report to ensure consistently high mapping rates across samples. The use of a splice-aware aligner like STAR ensured

that reads spanning exon–intron junctions were correctly mapped to the genome, an essential consideration for eukaryotic transcriptome data [28].

Gene-level quantification of aligned reads was carried out using FeatureCounts to generate gene expression levels. Subsequent data analysis, including normalization and differential expression, was performed using DESeq2 for all comparisons. P-values and log2 fold changes were generated using the WALD test. Volcano plots were created using GraphPad Prism (version 10.4.0; GraphPad Software, San Diego, CA, USA). For further analysis, genes with a p-value less than 0.05 and a log2 fold change greater than 1.5 were considered differentially expressed genes (DEGs). These DEGs were mapped using the Qiagen Ingenuity Pathway Analysis (IPA) (Redwood City, CA, USA) suite, and functional annotation and clustering were carried out using the Database for Annotation, Visualization, and Integrated Discovery (DAVID) (Bethesda, MD, USA). Sankey diagrams were created using SankeyMatic (Web-based tool; Steve Bogart).

RNA sequencing on average, generated 90,409,278 input reads per sample (Read 1 + Read 2). Of these, an average of 77,204,927 reads (85.40%) were uniquely mapped, while an average of 4.21% of reads mapped to multiple loci and 10.23% were unmapped due to being too short.

RNA sequencing data was deposited to NCBI GEO, accession number GSE301346.

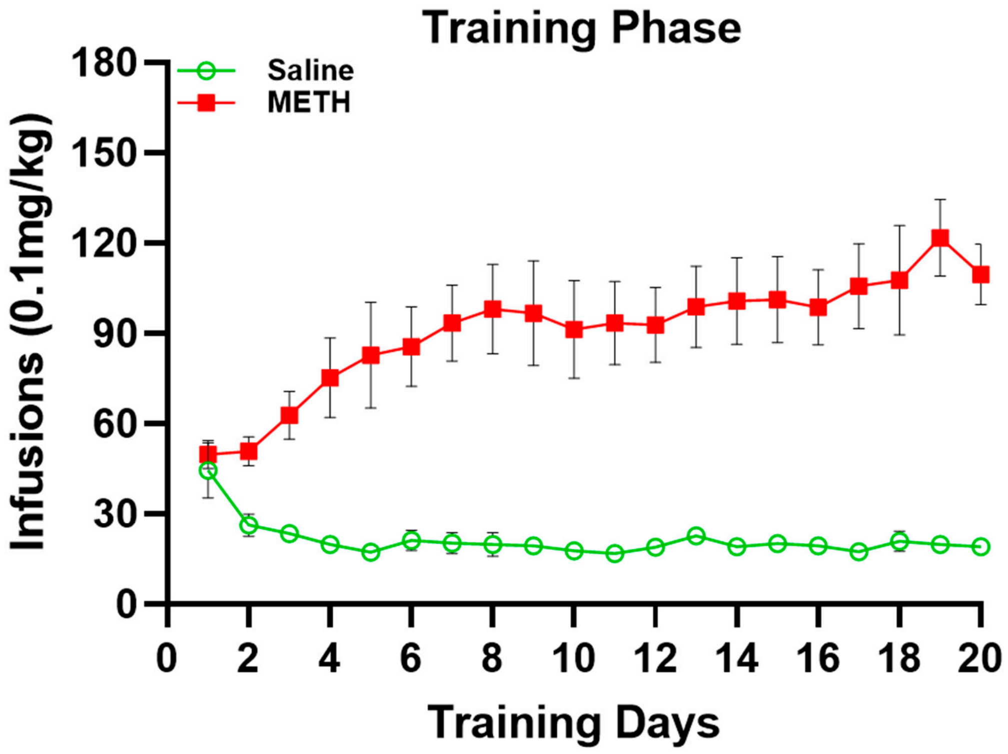

Figure S1. Training phase infusions of METH taking rats showing a steady significant rise in.METH infusions for the first 20 days.

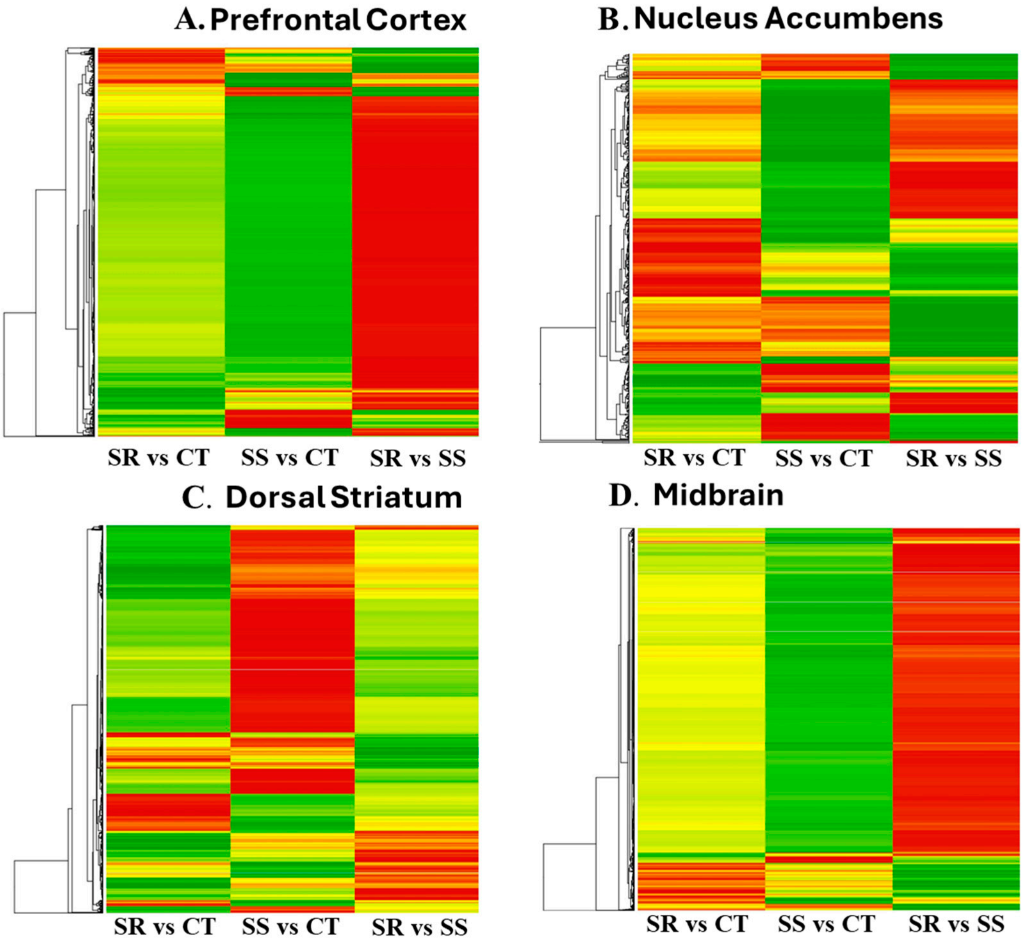

Figure S2. Heatmap analysis showing hierarchical clustering of all differential expressed genes (DEGs) across the SR vs CT; SS vs CT; and SR vs SS comparisons in the **(A)** prefrontal cortex, **(B)** nucleus accumbens, **(C)** dorsal striatum, and **(D)** midbrain. The color scale indicates the relative expression levels, with red representing up-regulation and green indicating down-regulation.

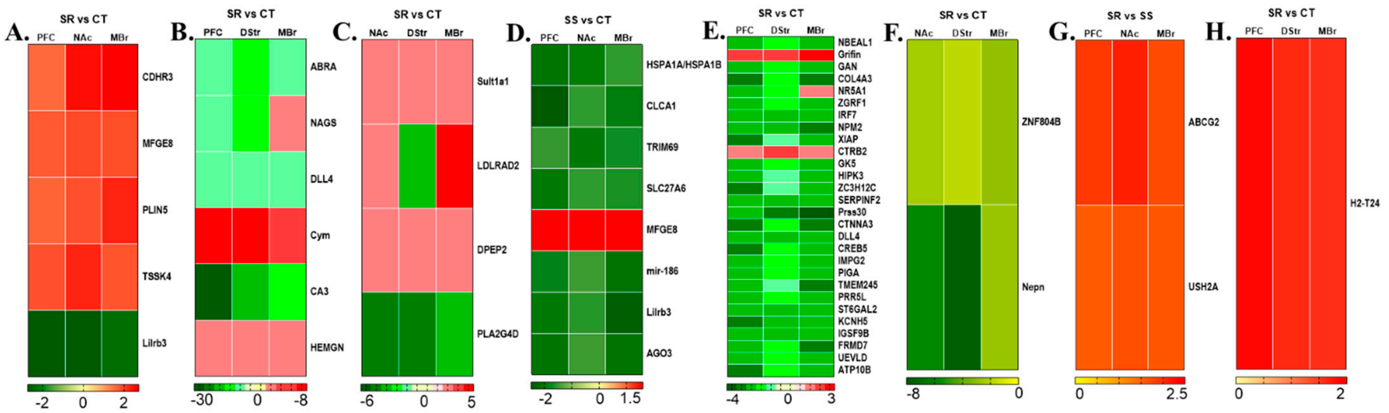

Figure S3. Heatmap analysis showing hierarchical clustering of differential expressed genes (DEGs) found to be shared across multiple brain regions. In the SR vs CT comparison, **(A)** 6 differentially expressed genes were found in the prefrontal cortex (PFC), nucleus accumbens (NAc), and midbrain (MBr); **(B)** 6 differentially expressed genes were found in the PFC, DStr and MBr; and **(C)** 4 differentially expressed genes were found in the NAc, DStr, and MBr. In the SS vs CT comparison, **(D)** 8 differentially expressed genes were found in the PFC, DStr, and MBr; **(E)** 28 differentially expressed genes were found in the PFC, DStr and MBr; and **(F)** 2 differentially expressed genes were found in the NAc, DStr, and MBr. In the SR vs SS comparison, **(G)** 2 differentially expressed genes were found

in the PFC, NAc and MBr; and **(H)**1 differentially expressed genes was found in the PFC, DStr, and MBr. The color scale indicates the relative expression levels, with red representing up-regulation and green indicating down-regulation.

Table S1. List of differential expressed genes (DEGs) shared across multiple brain regions, (A) SR vs CT (B) SS vs CT (C) SR vs SS. Green color indicate "Downregulated genes", whereas red color indicate "Upregulated genes".

A

SR vs CT				
Gene Name	PFC	NAc	DStr	MBr
NPAS4	-2.91	-2.36	-2.76	-2.52
FOS	-3.09	-2.18	-2.56	-1.88
AHSP	1.84	1.99	1.83	2.06
ATF3	-5.74	-2.21	-2.55	----
LTBP2	-1.57	-1.57	-1.93	----
EGR2	-3.11	-2.08	-2.21	----
NR4A1	-2.33	-1.84	-1.86	----
APOLD1	-3.50	-1.91	-2.10	----
PLAC8	2.20	2.24	2.62	----
CDHR3	1.55	2.48	----	2.72
MFGE8	1.75	1.91	----	1.87
PLIN5	1.63	1.84	----	2.28
TSSK4	1.82	2.27	----	1.81
Lilrb3	-2.73	-2.73	----	-2.39
ABRA	-1.72	----	-3.17	-2.14
NAGS	-1.55	----	-3.08	2.05
DLL4	-2.27	----	-1.82	-2.02
Cym	6.52	----	7.20	3.56
CA3	-29.51	----	-11.44	-8.62
HEMGN	2.52	----	2.51	1.82
Sult1a1	----	1.73	1.94	1.68
LDLRAD2	----	1.61	-1.53	4.87
DPEP2	----	1.54	1.81	1.82
PLA2G4D	----	-2.50	-2.74	-1.89

B

SS vs CT				
Gene Name	PFC	NAc	DStr	MBr
NPAS4	-1.98	-2.16	-1.71	-1.78
Cym	6.56	6.18	7.43	3.54
Tmem30c	-2.59	-2.77	-3.25	-2.43
TET1	-2.63	-1.68	-1.40	-2.14
USH2A	-2.36	-1.61	-1.51	----
EFCAB3	-1.70	-2.46	-1.98	----
SYT8	1.51	1.89	1.59	----
SCML4	-1.91	-1.58	-1.96	----
HSPA1A/HSPA1B	-2.07	-1.96	----	-1.54
CLCA1	-2.49	-1.53	----	-1.94
TRIM69	-1.54	-1.98	----	-1.69
SLC27A6	-2.04	-1.53	----	-1.65
MFGE8	1.58	1.60	----	1.58
mir-186	-1.86	-1.50	----	-2.09
Lilrb3	-2.00	-1.59	----	-2.41
AGO3	-2.07	-1.52	----	-2.11
NBEAL1	-1.72	----	-1.31	-1.55
Grifin	2.29	----	2.15	2.70
GAN	-1.88	----	-1.25	-1.91
COL4A3	-2.16	----	-1.30	-2.25
NR5A1	-1.56	----	-1.37	1.61
ZGRF1	-1.52	----	-1.26	-1.50
IRF7	-1.65	----	-1.60	-1.62
NPM2	-1.90	----	-1.85	-2.49
XIAP	-2.12	----	-1.21	-1.99
CTRB2	1.82	----	2.44	1.84
GK5	-1.61	----	-1.25	-1.54
HIPK3	-1.75	----	-1.15	-1.92
ZC3H12C	-2.20	----	-1.22	-1.89
SERPINF2	-1.61	----	-1.56	-1.63
Prss30	-1.54	----	-2.65	-4.63
CTNNA3	-2.37	----	-1.49	-2.05
DLL4	-1.89	----	-1.51	-1.82
CREB5	-2.31	----	-1.33	-1.66
IMPG2	-1.80	----	-1.32	-1.70
PIGA	-1.64	----	-1.31	-1.58
TMEM245	-1.84	----	-1.21	-2.04
PRR5L	-1.59	----	-1.43	-1.51
ST6GAL2	-1.68	----	-1.86	-1.80
KCNH5	-2.00	----	-1.86	-1.74
IGSF9B	-1.92	----	-1.53	-1.67
FRMD7	-1.52	----	-1.38	-2.03
UEVLD	-1.91	----	-1.29	-1.86
ATP10B	-2.80	----	-1.45	-1.80
ZNF804B	----	-2.46	-1.77	-2.98
Nepn	----	-5.69	-7.66	-2.74

C

SR vs SS				
Gene Name	PFC	NAc	DStr	MBr
IRF7	1.76	1.77	1.91	1.94
ABCG2	1.90	2.12	----	1.73
USH2A	1.58	1.71	----	1.62
H2-T24	1.87	----	1.64	1.58
